# Edible Insects versus Meat—Nutritional Comparison: Knowledge of Their Composition Is the Key to Good Health

**DOI:** 10.3390/nu13041207

**Published:** 2021-04-06

**Authors:** Agnieszka Orkusz

**Affiliations:** Department of Biotechnology and Food Analysis, Wroclaw University of Economics and Business, 53-345 Wroclaw, Poland; agnieszka.orkusz@ue.wroc.pl; Tel.: +48-(71)-3680480

**Keywords:** edible insects, meat, diet, nutritional value, human health, environmental concern

## Abstract

Recently, attention has been drawn to the fact that increasing the consumption of edible insects may positively impact the state of the natural environment and reduce the problem of malnutrition in large parts of society. Indeed, insects are seen as an alternative to traditional meat products, primarily meat. This article aimed to compare the nutritional value of edible insects and meat. Based on tables of composition and nutritional value and on the licensed computer program Diet 6D, data on the nutritional value of 10 commonly consumed meat types were compiled. Based on a literature review, data on the nutritional value of seven commercially available edible insect species were collected and collated. There was a comparison of 100 g of edible insects with 100 g of meat (fresh weight). In addition, the atherogenic index thrombogenic index, the hypocholesterolemic/hypercholesterolemic ratio, and the nutritional quality index were calculated. It was found that both meat and insects are rich in nutrients, including those considered essential for the proper development and functioning of the human body. At the same time, it has been shown that the content of individual nutrients in both insects and meat varies significantly.

## 1. Introduction

Food security is predicted to be exposed to stress in the coming decades due to rapid global population growth and rising animal protein demand. It is estimated that by 2050, the global population will reach about 9.8 billion [1,2,3], and the demand for food will increase by 60% [4]. Increased livestock production requires an expansion of agricultural areas, increased water and heat consumption, and animal feed consumption. However, this is not possible due to the reduction in arable land and declining freshwater supplies. Increasing meat production is recognized as one of the leading causes of climate change, threatening future generations’ welfare. Livestock is estimated to be responsible for 20% of global greenhouse gas emissions [5]. Large livestock farms can trigger epidemics such as bird flu and swine flu. There is also much talk about the suffering of mass-slaughtered animals. In this context, it makes sense to look for new sources of nutrients, including protein.

There are alternatives to meat on the market, from tofu to seitan, but the production of these meat substitutes requires a lot of processing. This raises a question about how wholesome these products are. Producing soy protein isolate, a common ingredient in plant-based meat alternatives, is less environmentally friendly than meat production due to the large amounts of water and fossil fuel energy required to extract the protein isolate from soybean meal [6].

Edible insects are a unique food ingredient with great potential to contribute to global food security, and provide an interesting food alternative, especially to meat. The use of insects in food production is also essential to mitigate the negative effects of climate change [7]. Edible insects, considered as a protein source for humans, produce much fewer greenhouse gases and require much less land than conventional livestock (e.g., chickens, pigs, and cattle) [8]. Insects have a high food conversion ratio to produce the same amount of protein, e.g., crickets need six times less feed than cattle, four times less than sheep, and half that of pigs and broiler chickens [9].

In addition to the benefits of introducing edible insects into the human diet, the potential food safety hazards are also indicated, which are classified into three categories: allergens, biological hazards, and chemical hazards [7]. The scientific literature on edible insects’ food safety aspects is limited. Other authors pointed out that some types of proteins present in edible insects, including arginine kinase, are a potential allergen source. Apart from aginine kinase, other common allergens linked to edible insects include α-amylase and tropomyosine [7,10]. Insects meant for human consumption must come from a reliable source. Insect producers are required to conform to all existing regulations for food production. Edible insects should be grown under hygienic conditions and regularly tested for quality like any other foods [11].

More than 2000 edible insect species are consumed around the world [9,12]. The most commonly consumed group of insects are beetles; caterpillars; bees, wasps, ants; grasshoppers, locusts, and crickets; true bugs; dragonflies; termites; flies; cockroaches; spiders, and other orders (Table 1).

Interest in entomophagy is growing in the West. The United Nations Food and Agriculture Organization (FAO) is largely responsible for popularizing edible insects’ introduction into the human diet. In 2013, the FAO published an extensive report [9] outlining the arguments for why Westerners should accept this new type of food. In Regulation 2015/2283 [13] of the European Parliament and the Council of the European Union, whole insects and their parts were included in the category of novel foods.

Furthermore, in 2015, the European Food Safety Authority (EFSA) provided a scientific opinion on insect consumption and suggested a list of insect species with high potential for use as food for both humans and animals [14]. In 2021 EFSA gave a positive scientific opinion on the safety of dried yellow mealworm (*Tenebrio molitor* larva) as a novel food according to Regulation (EU) 2015/2283 [15]. It is worth noting that despite international agencies such as the FAO and EFSA, which advocate the nutritional, environmental, and economic benefits of entomophagy, attitudinal barriers still persist in Western societies [16,17]. Before insects become a large scale food product for humans worldwide, the Western world must overcome culturally determined aversion. Most importantly, insects should be turned into an appealing product [11].

It is well known that meat is a source of complete protein, easily digestible iron, B vitamins, and fat. Due to its high content of saturated fatty acids and heme, ruminant meat is considered less healthy than other species. Consumption of red meat (meat from mammals such as cows, calves, pigs, sheep, and horses) is associated with a higher risk of stroke, diabetes, and colorectal and lung cancer [18]. According to the American Institute for Cancer Research, eating more than 550 g of red meat per week may increase cancer risk. However, there is insufficient evidence to link white meat (poultry, domestic rabbit) to chronic diseases [19,20,21,22,23,24].

Compared to meat from slaughtered animals, standardized data on edible insects’ nutritional value are limited and inconclusive [25,26,27,28,29]. These data concern different species of insects and their development stages, diet, and nutrients, and are presented in different units.

Moreover, in the articles on the nutritional value of edible insects, the data are mainly based on dry matter, which cannot be directly used to assess human nutrition [28,29,30]. This is because the assessment of the nutritional value of food is carried out based on standardized tables of the composition and nutritional value of food products, which contain data on the nutrient content of food products in the form of fresh mass.

This limited information is used to justify generalized claims about edible insects’ health benefits as a single homogeneous food category [27], which is seriously considered as an alternative to meat.

Therefore, the study aimed to: (1) collect and compile, based on a literature review, the nutritional data (expressed as fresh weight) of selected species of edible insects. The choice of insect species was dictated by the largest amount of literature data describing their nutritional value to include the greatest possible amount of nutrients. All data was unified per 100 g edible portion (fresh weight); (2) summarize the nutritional value of 10 different types of meat from slaughtered animals based on the tables of composition and nutritional value (based on the program Diet 6D); (3) compare the composition of edible insects and meat in terms of energy value, protein, fat, fiber, cholesterol, amino acids, fatty acids (saturated, monounsaturated and polyunsaturated), minerals (Na, K, Ca, P, Mg, Fe, Zn, Cu, Mn, I), and vitamins (A, E, B_1_, B_2_, B_3_, B_6_, B_12_, C). There was a comparison of 100 g of edible insects with 100 g of meat (fresh weight). In addition, the atherogenic index (AI), thrombogenic index (TI), and the hypocholesterolemic/hypercholesterolemic ratio (h/H) were calculated based on the fatty acids profile. These indices serve as predictors of cardiovascular risk. The nutritional quality index (INQ) was also used to assess edible insect and meat nutritional value. It expresses the degree to which the consumed product meets the energy needs of a person and satisfies their need for a specific nutrient.

Comparing the composition of edible insects and meat will indicate which species of insects and meat have high nutritional value and are the best sources of nutrients, including complete protein, essential amino acids, unsaturated fatty acids, minerals, and vitamins. Presenting data on edible insects as standardized values in the form ‘per 100 g edible portion on a fresh weight basis’ can make the results easily understood not only by policymakers and nutritionists but also by consumers. This will make it easier to compose a balanced diet in which edible insects can replace meat. This comparison will therefore help to bring closer the perception of insects as a new food ingredient alternative to meat in particular.

## 2. Materials and Methods

### 2.1. Edible Insects Species Selection

The edible insect species selected for the comparison were as follows: house cricket larvae and adults (*Acheta domesticus*), field crickets (*Gryllus bimaculatus* adult), mealworm larvae and adults (*Tenebrio molitor*), superworm larvae (*Zophobas morio*), waxworm larvae (*Galleria mellonella*), silkworm larvae (*Bombyx mori*), and mopane caterpillar larvae (*Gonimbrasia belina*), which are now commercially available worldwide [9,20,31,32]. These are species collected in the wild as well as cultured on a large scale and sold commercially for use as food and feed. At the same time, these species attract the greatest attention of scientists. Thus, for the selected insect species, based on the literature data, it was possible to collect and collate data on the nutrients per 100 g edible portion in terms of fresh weight.

### 2.2. Meat Species Selection

The following meat species were selected for comparison: mutton leg, veal leg, horse meat, pork shoulder, beef sirloin, rabbit carcass, goose and duck carcass, and turkey and chicken breast and drumstick. These are some of the most widespread meat species globally, which constitute an essential part of the human diet. These species differ in their composition. This is important because the study compares meat and edible insects’ nutritional values, with insects being considered an alternative food ingredient to meat. Among the analyzed types of meat, some species are classified as white meat (galloping poultry (chickens, turkeys), waterfowl (geese, ducks), and rabbits) or red meat (cows, calves, pigs, sheep, and horses). This division depends on the concentration of myoglobin in the muscles of the animals. Red meat owes its dark red color to the presence of a high concentration of myoglobin, and the heme content of red meat is 10-fold higher than that of white meat [33,34].

### 2.3. Data Quality 

An extensive literature search was conducted from October to December 2020 via the Science Direct and Scopus databases. The following keywords were used: edible insects/adult insect/larva/*Acheta domesticus*/*Tenebrio molitor*/*Gryllus bimaculatus*/*Zophobas morio*/*Mopane caterpillar*/*Bombyx mori*/*Galleria mallonella*/silkworm/waxworm/nutritional value/amino acids/fatty acids/minerals/vitamins. Insect composition data were collected from scientific articles and from the INFOODS database [35] and the Thai food composition database [36].

Different meats’ nutritional values were compiled based on the licensed computer program Diet 6D, containing tables of composition and nutritional values of food products [37]. Diet 6D was developed at the Independent Laboratory of Epidemiology and Nutrition Standards, Institute of Food and Nutrition, Warsaw, Poland, in 2018. This program calculates the nutritional value and composition of consumed products, foods, and thus planning diets.

### 2.4. Dietary Indicators

Atherogenic index (AI) and thrombogenic index (TI) [38] were calculated according to the following equations:

AI = (C12:0 + 4 × C14:0 + C16:0)/(MUFA + *n*-6 + *n*-3)

TI = (C14:0 + C16:0 + C18:0)/ (0.5 × MUFA + 0.5 × *n*-6 + 3 × *n*-3 + *n*-3/*n*-6)

The proportion of fatty acids with a hypocholesterolemic effect (h), i.e., cholesterol-lowering, and a hypercholesterolemic effect (H), i.e., cholesterol-elevating (hypocholesterolemic/hypercholesterolemic ratio–h/H) [39,40], was also calculated according to the equation:

h/H = (C18:1 c9 + C18:2 *n*-6 + C18:3 *n*-3 + C20:3 *n*-6 + C20:4 *n*-6 + C20:5 *n*-3 + C22:5 *n*-3)/(C12:0 + C14:0 + C16:0)

### 2.5. Nutritional Quality Index

The nutritional quality index (INQ), which is a measure of food density, was calculated for nutrients according to the following equation [41]:

$$\mathrm{INQ}=\frac{\text{ingredient content per 100 g of product}\times \text{energy requirement standard}}{\text{energy value per 100 g of product}\times \text{requirement standard for ingredient}}$$

The values of the INQ index for protein, selected minerals (Na, K, Ca, P, Mg, Fe, Zn, Cu, Mn, I), and vitamins (A, E, B_1_, B_2_, B_3_, B_6_, B_12_, C) were calculated according to the requirements of a premenopausal woman aged between 31 and 50 years, body weight 45 kg, and moderate physical activity. The values of the daily recommended intake of nutrients come from the Polish standards developed in 2020 by the experts of the National Institute of Public Health–National Institute of Hygiene Institute [42]. Calculations were performed in a Microsoft Excel spreadsheet. 

### 2.6. Ethical Statement

Ethical review and approval were waived for this study due to the research being based on a review of the published literature and secondary data obtained from the licensed computer programme Diet 6D (Institute of Food and Nutrition, Independent Laboratory of Epidemiology and Nutrition Standards, Warsaw, Poland). 

## 3. Results

The energy values and the elemental chemical composition of various types of meat and insects are presented in Table 2.

The energy value of meat and insects varied depending on their species, the type of muscle, and the stage of insect development, and ranged from 83 to 199 kcal/100 g in meat and from 120 to 274 kcal/100 g in insects (Table 2). The least caloric meats were turkey and chicken breast and veal leg, and the most caloric were mutton leg and duck carcass. Insect larvae were generally higher in calories than adult insects.

In terms of protein content, both adult forms of the *Tenebrio molitor* species (24.13 g/100 g) and the larval forms of the species *Bombyx mori* (23.1 g/100 g), *Tenebrio molitor* (25.0 g/100 g), and *Gonimbrasia belina* (35.2 g/100 g) contained the highest protein content. Meat had a lower protein content than edible insects. Among the analyzed types of meat, poultry breast muscles, beef sirloin, and horse meat (19.2–21.5 g/100 g) had a high protein content.

The data presented in Table 2 show that the fat content varied greatly both in meat from different animal species and in insects. The fat content ranged from 0.7 to 18.30 g/100 g in meat, while in insects, it ranged from 4.4 to 24.9 g/100 g. Among the compared meat species, the highest fat content, per 100 g edible portion, was found in the carcasses of waterfowl (goose, 13.04 g; duck, 18.30 g) and mutton leg (15.12 g). In the case of insects, insect larvae contained more fat than adult forms, except for the larvae of *Acheta domesticus* (Table 2).

The meat of slaughtered animals, unlike edible insects, lacked dietary fiber (Table 2).

Table 2 shows that the highest cholesterol content was found in adult insects of *Acheta domesticus* (98.5 mg/100 g) and *Gryllus bimaculatus* (195 mg/100 g), while the lowest was in goose carcass (32.8 mg/100 g). The cholesterol content of insect larvae was similar to that of veal leg, pork shoulder, rabbit carcass, duck carcass, and turkey breast.

The nutritional value of a product can be determined by its nutritional density, which is measured by the nutritional quality index (INQ). The nutritional density of a product depends on its unique nutrient content and energy content. An INQ value of around 1.0 means that the product is well-balanced in terms of the component and energy content. A value below 1.0 indicates that the product does not provide the appropriate amount of nutrients. A value higher than 1.0 indicates that the product has a very high nutrient to energy content.

Table 2 gives the value of nutritional quality indices for the protein of various insect species and meats. The INQ values indicated that the protein content of both insects and meat was well-balanced. The INQ ratios for protein exceeded the value of 1.0. The highest INQ values were found in the larvae of *Gonimbrasia belina* insects and in turkey and chicken breast muscles (Table 2). Therefore, these species may be the best dietary component for supplementing the daily food ration in which there is a protein deficiency.

The nutritional value of protein is determined by its essential amino acid content. The level of amino acids, including essential amino acids, in the meat of different animal species and edible insects, is presented in Table 3. The data therein indicate that both meat and edible insects are sources of complete animal protein containing all essential amino acids in its composition. *Bombyx mori* and goose carcasses are the insect and meat species, respectively, with the lowest levels of all essential amino acids (Table 3).

The profile of fatty acids in the lipids of meat and insects is presented in Table 4 and Table 5.

Among the saturated fatty acids in the lipids of both meat and insects (Table 4 and Table 5), the highest share was palmitic acid (C 16:0), and was followed by stearic acid (C 18:0). The predominant monounsaturated fatty acid was oleic acid (C 18:1). The presence of linoleic acid (C 18:2 *n*-6), which belongs to essential fatty acids, was particularly important. Linoleic acid content was much higher in insects of species such as *Acheta domesticus*, *Tenebrio molitor*, *Zophobas morio*, and *Gonimbrasia belina* in comparison to meat.

The ratio of polyunsaturated to saturated fatty acids was a significant indicator of fat quality, and its recommended amount in the diet should be higher than 0.40. Therefore, from the point of view of human nutrition, the fat from such insect species as *Acheta domesticus*, *Tenebrio molitor*, *Zophobas morio*, and *Gonimbrasia belina* has a more favorable PUFA/SFA ratio than meat, except for chicken breast and drumstick (Table 4 and Table 5). 

The marked fatty acid indexes (AI, TI, and h/H) may indicate the direction of the consumed lipids’ impact and provide a better assessment of foods’ nutritional quality than only the sum of SFA, MUFA, PUFA, and their ration. The index of atherogenicity suggests a relationship between saturated (pro-atherogenic) fatty acids, favoring lipids’ attachment to endothelial cells of the circulatory system, and unsaturated (antiatherogenic) fatty acids, which reduce cholesterol levels and prevent the occurrence of coronary artery diseases. The thrombogenicity index shows the tendency for blood clots to form in the blood vessels. Like the atherogenicity index, it is expressed by the ratio of saturated to unsaturated fatty acids; however, occurring in different proportions.

From the nutritional point of view, the most favored indicators, including the weakest athero- and thrombogenic effects and the most favored proportion of hypo- and hypercholesterolemic acids, are found in goose carcass and *Tenebrio molitor* larvae (Table 4 and Table 5).

Meat and insects are important sources of minerals (Table 6). The contents of minerals in both meat and insects are very diverse. Insects, regardless of the species and form of development, are characterized by higher calcium, zinc, copper, and manganese contents than meat (Table 6). It is well known that meat is an essential source of easily digestible iron from heme pigments, i.e., myoglobin and hemoglobin. Large animal meat (beef sirloin, horse meat) contains more iron (3.1–3.5 mg/100 g) than poultry meat (0.4–1.3 mg/100 g). The lowest iron content among the presented insect species was found in *Zophobas morio* larvae (1.99 mg/100 g), while the highest was in *Gonimbrasia belina* larvae (51.05 mg/100 g) (Table 6).

Table 7 shows the INQ indexes of minerals for selected insect species and meats. Despite the significant content of minerals in the selected types of meat, only the content of phosphorus, zinc, and copper (except for mutton leg and chicken breast) was well-balanced. In insects, the content of most minerals (phosphorus, magnesium, iron, zinc, copper, and manganese) was well-balanced with energy (Table 7). Adult insects of the species *Acheta domesticus* and *Gryllus bimaculatus* and the larva of the species *Gonimbrasia belina* can be used to supplement a daily food ration that is deficient in Na, K, Ca, P, Mg, Fe, Zn, Cu, and Mn.

The vitamin content in both insects and meat varies significantly (Table 8). Among the analyzed species of insects and meat, pork (0.49 mg/100 g) and insects of the species *Gryllus bimaculatus* (0.36 mg/100 g) and *Bombyx mori* (0.33 mg/100 g) had the highest thiamine content. However, a lower content of this vitamin is observed in other types of meat, including the carcasses of rabbits and poultry, and insects. The thiamine content was in the range of 0.02–0.49 mg/100 g in meat and 0.04–0.36 mg/100 g in insects. Insects, regardless of the species and form of development, were characterized by a higher content of tocopherol, riboflavin, and vitamin C than meat (Table 8). Both insects and meat were good sources of niacin; chicken breast contained the highest content (12.44 mg/100 g). Meat has quite varied cobalamin content (Table 8). This vitamin is not found in plants. It is considered to be a regulator of proteins’ biological value due to its participation in amino acid metabolism. An excellent source of cobalamin was rabbit carcass (7.90 µg/100 g) and horse meat (3.10 µg/100 g). This vitamin was found in turkey drumstick, beef sirloin, and veal leg in a smaller amount (at the level of 1.03–1.70 µg/100 g). The poorest sources of this vitamin (at the level of 0.12–0.13 µg/100 g) were waterfowl carcasses (Table 8). The cobalamin content in insects ranged from 0.01 µg/100 g (*Bombyx mori*) to 0.99 µg/100 g (*Zophobas morio*).

Table 9 indicates the value of nutritional quality indicators for vitamins in selected insect species and meats. In insects, regardless of species and form of development, the content of most vitamins (retinol, thiamine (except in the adult insects of the species *Acheta domesticus*), riboflavin, niacin, and pyridoxine) was well-balanced to energy (Table 9). INQ ratios exceed the value of 1.0.

## 4. Discussion

The nutritional value of food is determined by both its chemical composition and the proportions between the ingredients and their bioavailability, i.e., their use in building cells and tissues as well as vital functions of the body. Therefore, it is primarily about the high content of balanced protein, determined by the amino acid composition, the content and composition of lipids, and the content of vitamins and minerals.

Comparing the nutritional value, it was found that both meat and insects are rich in nutrients (protein, fat, minerals, vitamins), including those considered essential (essential amino acids, polyunsaturated fatty acids) for proper development and functioning of the human body (Table 2, Table 3, Table 4, Table 5, Table 6, Table 7, Table 8 and Table 9). At the same time, it was observed that the content of individual nutrients varies significantly. This variation was expected because it is well known that the nutritional value of the meat of slaughtered animals varies depending on the species, age, sex, muscle type, environmental conditions of rearing, and diet [51,52]. On the other hand, the nutritional value of insects depends on their developmental stage [53]; sex [54,55,56]; feed [57,58]; and the way they are reared, prepared, and processed [59]. Adult insects are characterized by lower caloric content than the larvae and pupae [60], which is related to the amount of fat. A higher content was observed in larvae and pupae than in adults [61]. The insects’ adult forms were characterized by the highest protein content, followed by the larvae and pupae [60]. Females had a higher energy value and contained significantly more lipids and less protein than males [56]. Sex differences in nutrients have been attributed to differences in their nutritional requirements for optimal performance and maximization of fitness, which are known to be sex-specific [62].

The nutritional quality index shows that both insects’ and meat’s protein content is well-balanced (Table 2). Among the analyzed species of insects and the meat of slaughtered animals, the best component of the diet supplementing the daily food ration with protein deficiency are the larvae of insects of the species *Gonimbrasia belina* and the breast muscles of turkeys and chickens (Table 2). Additionally, adult forms of the species *Tenebrio molitor* (24.13 g/100 g) and the larval forms of the species *Bombyx mori* (23.1 g/100 g), *Tenebrio molitor* (25.0 g/100 g), and *Gonimbrasia belina* (35.2 g/100 g) were characterized by a higher protein content than meat (Table 2). Edible insects can therefore be widely used in the food industry. They can be used to enrich diets, especially a plant-based diet based on cereal proteins that is poor in essential amino acids such as lysine, threonine, and tryptophan, with complete proteins. They can also form the basis of high-protein products. They must be incorporated into the diet in a ground form, invisible to the consumer, because they arouse disgust among Europeans [16,17].

Lipids are the main and most concentrated source of energy in human food. The nutritional value of lipids is determined by the quantitative and qualitative composition of fatty acids. Among saturated fatty acids, palmitic acid content (C 16:0) was the highest in both meat and insects. It is included in the group of acids which are believed to have a negative effect on human body, especially by stimulating blood cholesterol—its LDL fraction in particular [63,64]. Among monounsaturated and polyunsaturated acids, the largest amount, in both meat and insects, was found in oleic (C 18:1) and linoleic acid (18:2 *n*-6). The positive effect of these acids on the human body has been noticed many times [65,66].

A useful indicator of the nutritional value of a food is the *n*-6/*n*-3 ratio. Simopoulos and De Meester [67] consider the optimal ratio of *n*-6/*n*-3 in the diet to be the proportion typical of primitive societies (1:1), while currently, in the diets of people in various countries of the world, it ranges from 4:1 to 25:1 [68]. The *n*-6/*n*-3 ratio significantly deviates from the recommended value due to too high consumption of *n*-6 fatty acids and foods containing saturated fatty acids [67].

Experts of the American Institute for Cancer Research and the World Cancer Research Foundation point at the opposite role of *n*-3 PUFA and *n*-6 PUFA in the development of the neoplastic process, and prove that the influence of lipids on cancer formation depends on the mutual proportion (disproportion) of fatty acids from the *n*-3 and *n*-6 family [69]. The *n*-6 PUFAs act pro-cancer and the *n*-3 PUFAs act anti-cancer. Polyunsaturated fatty acids act at the promotion stage (proliferation of neoplastic cells and reproduction of genetic changes in daughter cells) of the neoplastic process [69]. Excessive consumption of *n*-6 PUFA with a low consumption of *n*-3 PUFA favors transformations of *n*-6 acids and, additionally, increases the disproportion of these acids in the body to the detriment of *n*-3 PUFA [69]. Such a change in the *n*-6/*n*-3 PUFA ratio in the diet is responsible for the persistence of the pro-inflammatory state and, consequently, for the tendency to permanent inflammation in the body [67].

The *n*-6/*n*-3 ratio varies significantly in meat and insects (Table 4 and Table 5).

The content of *n*-6 family acids, compared to *n*-3 family acids, in insects and meat ranges from 0.43 to 19.25 and 2.02–17.73, respectively. Among the listed meat species, the highest *n*-6/*n*-3 ratio was found in the meat of burrowing poultry (except for chicken breast), and in the case of insects, in adults of the species *Acheta domesticus* and the larvae of the species *Tenebrio molitor*. It is therefore essential to diversify the diet and enrich it with products containing *n*-3 polyunsaturated fatty acids. One should also be aware that the fat of selected species of insects and meat, in which unsaturated fatty acids have a significant share, is more susceptible to oxidation processes unfavorable not only for taste but also for health [70,71].

Depending on the species, the meat of slaughtered animals and insects contain a varied amount of minerals and vitamins necessary for the human body’s proper functioning (Table 6 and Table 8). This knowledge can be beneficial for balancing the content of minerals and vitamins in daily food rations in which there are irregularities related to their insufficient content. The minerals that are assigned a unique role in the proper development and functioning of the human body are calcium and iron [72,73].

Too low calcium content in the diet is a disadvantageous phenomenon because, in the case of nutritional deficiencies of calcium, its supplementation takes place at the expense of bone tissue, causing an increase in the pace of bone mass density decline. This may lead to, among other things, spine deformities, body decalcification, tooth damage, and increased risk of osteoporotic changes [42,74]. Dietary calcium deficiency also influences the development of cardiovascular diseases [75]. Insufficient calcium intake may also increase the risk of stroke in middle-aged women [76]. Calcium in the body has anti-inflammatory and anti-allergic effects [42,77].

At an early stage, insufficient intake of iron manifests itself in the form of changes in the mucous membranes of the mouth and esophagus. Iron deficiency is often accompanied by headaches and dizziness, fatigue, decreased activity, and an inability to focus [78]. Iron deficiency is one of the most common causes of anemia in the elderly [79].

The adult insects of *Acheta domesticus* and *Gryllus bimaculatus* and the larva of the species *Gonimbrasia belina* can be used to supplement a daily ration that is deficient in both calcium and iron (Table 7). Importantly, to use the content of nutrients in insects, they must be eaten whole. Taking into consideration consumers’ reluctance to eat insects in their visible form, it is recommended to grind them before adding to traditionally eaten dishes.

Vitamins play a regulatory role in the body and determine the human body’s development, health, and physical performance [80]. The meat of slaughtered animals and insects differs in terms of the presence and contents of vitamins. Meat and insects are primarily a source of water-soluble vitamins of the B group. The level of fat-soluble vitamins, i.e., vitamin A and E, is much lower. Meat, in contrast to insects, does not contain vitamin C (Table 8). Considering the nutritional quality index values for vitamins (Table 9), both the meat of slaughtered animals and insects should be regarded as well-balanced in terms of B vitamins’ content.

Optimization of B vitamins’ intake is particularly important in people with disorders of folate metabolism due to genetic characteristics, particularly in people with mutations in the gene encoding the enzyme methylenetetrahydrofolate reductase (MTHFR) [42,81], and also in the elderly. This is because, with age, you can notice the deterioration in absorption and a reduced intake of vitamins with the diet. Maintaining an adequate level of B vitamins in the blood, especially riboflavin, among the elderly, positively affects their mental health and reduces the risk of cognitive impairment [82,83]. A diet rich in B vitamins, especially thiamine, riboflavin, niacin, cobalamin, and zinc, the presence of which is high in both insects and meat (Table 8 and Table 9), reduces the risk of non-alcoholic fatty liver disease [84].

Compared to meat, insects are a better dietary component for supplementing daily rations deficient in riboflavin (Table 9). The highest values of nutritional quality indicators for riboflavin were observed for the species *Acheta domesticus* (adult and larval form) and *Gryllus bimaculatus* (Table 9). Human beings are not able to synthesize riboflavin, and have to supply it to the body with food [85]. Therefore, it is justified to introduce insects to the human diet as an alternative source of many nutrients, including riboflavin, especially to groups of people at risk of riboflavin deficiency, i.e., those who consume excessive amounts of alcohol, and the elderly [86]. Symptoms of riboflavin deficiency are characterized by inflammation of the corners of the mouth and tongue, exfoliation of the epidermis, seborrheic dermatitis, and redness and dryness of the conjunctiva, but can also cause dysfunction of the nervous or endocrine system [87,88].

Meat (mainly horse meat, rabbit carcass, or turkey drumstick) can help supplement a daily food ration that is deficient in cobalamin (Table 9). Vitamin B_12_ deficiency in the body is a threat to people who have type 2 diabetes [89], stomach diseases (peptic ulcer disease, gastroesophageal reflux disease), or intestinal conditions (Whipple’s disease, Zollinger–Ellison syndrome, Crohn’s disease, celiac disease, ulcerative colitis) [90]. Perhaps edible insects, which are a good source of vitamins, including cobalamins, will gain recognition among people who eliminate meat from slaughtered animals from their diet.

It should be mentioned that the data on the nutritional value of edible insects were obtained from the literature review, while those referring to meat were from the tables of composition and nutritional value. The tables’ values are average data, so the nutrient content of a particular product may differ from the values given in the tables, as it depends on many factors, both genetic and environmental.

## 5. Conclusions

High protein content, which includes all essential amino acids, the presence of unsaturated fatty acids, vitamins, and minerals, make both insects and meat highly nutritious.

The comparative analysis carried out on the composition of the meat of slaughtered animals and insects cannot conclude unequivocally that insects have a higher nutritional value, because the content of individual nutrients varies significantly in both meat and insects. However, it can be seen that some of the analyzed edible insect species have a higher energy value than meat from slaughter animals and higher content of protein, fat, polyunsaturated fatty acids, and cholesterol. However, the content of saturated fatty acids, monounsaturated fatty acids, thiamine, niacin, cobalamin, and iron is lower. Insects, regardless of the species and form of development, are characterized by a higher content of tocopherol, riboflavin, calcium, zinc, copper, and manganese than meat. Contrary to meat, they are also a source of vitamin C and dietary fiber.

Knowledge of nutrients and the amounts in which they occur in various kinds of meat and species of insects can be used, among other things, to plan and prepare meals with the correct contents of nutrients. It may foster a change in eating habits and lead to a general improvement in human health. This knowledge can enrich the daily ration of those nutrients whose supply is too low in comparison to recommended values.

The acquired knowledge indicates that edible insects are a valuable food product. Their widespread use in the human diet may help solve the problem of global malnutrition.

## Figures and Tables

**Table 1:** Most commonly consumed insects around the world [12].

Group of Insects	Order	Number of Species	Number of Recorded Edible Insect Species per Group in the World	Percent of Recorded Edible Insect Species per Group in the World
Beetles	Coleoptera	370,000	659	31.2
Caterpillars	Lepidoptera	165,000	362	17.1
Ants, Bees, Wasps	Hymenoptera	198,000	321	15.2
Grasshoppers, Locusts, Crickets	Orthoptera	20,000	278	13.2
True bugs	Hemiptera	82,000	237	11.2
Dragonflies	Odonata	5500	61	2.9
Termites	Isoptera	2750	59	2.8
Flies	Diptera	122,000	37	1.8
Cockroaches	Blattodea	4000	37	1.8
Spiders	Araneae	40,000	15	0.7
Others	-	33,164 +	45	2.1

“+” more than.

**Table 2:** The basic nutrients content of edible insects and meat expressed per 100 g of edible portion [5,28,29,30,35,36,43,44,45,46].

Basic Nutrients Species	Energy (kcal/100 g)	Protein (g/100 g)	Fat (g/100 g)	Fiber (g/100 g)	Cholesterol (mg/100 g)	INQ Values for Protein *
*Acheta domesticus* A [28,29,44]	153	20.5	5.06	4.6	98.5	6.73
*Acheta domesticus* L [30,43,44,45]	137.5	15.4–17.5	4.4–7.9	2.3	-	6.13
*Gryllus bimaculatus* A [5,35,36]	120	15.75	5.5–5.75	3.4	195	6.72
*Tenebrio molitor* A [5,30,45]	178	24.13	6.14	7.4		6.94
*Tenebrio molitor* L [29,43,45,46]	247	25.0	12.91	3.52	51.3	5.18
*Zophobas morio* L [43]	206.9	18.6	14.4	3.78	45	4.60
*Gonimbrasia belina* L [28,35]	161	35.2	15.2	-	-	11.20
*Bombyx mori* L [5,35,45]	171.27–229	17.9–23.1	4.26–5.0	1.0–22.22	-	5.72
*Pyralidae* L [35,43,44,45]	274.7	16.1	24.9	2.1–3.4	75.3	3.00
Mutton leg	196.56	15.12	15.12	-	65.52	3.94
Veal leg	85.32	15.72	2.45	-	56.09	9.44
Horse meat	109	21.5	2.5	-	75	10.10
Pork shoulder	13.2	16.89	7.05	-	50.02	6.59
Beef sirloin	112	20.1	3.5	-	59	9.19
Rabbit carcass	123.24	16.59	6.32	-	51.35	6.89
Goose carcass	140.63	5.78	13.04	-	32.8	2.11
Duck carcass	199.04	8.64	18.30	-	48.64	2.22
Turkey breast	83	19.2	0.7	-	49	11.85
Turkey drumstick	100	16.6	3.7	-	81	8.50
Chicken breast	98	21.5	1.3	-	58	11.24
Chicken drumstick	125	17.8	6	-	84	7.29

* INQ values were calculated according to a daily requirement of 41 g of protein for a woman (31–50 years, 45 kg body weight, and moderate physical activity, energy demand 2100 kcal/day [42]). A—adult insect; L—larval form.

**Table 3:** Amino acid composition data of edible insects and meat (mg/100 g edible portion) [28,35,43,44,45,47,48].

	Essential Amino Acids									Non-Essential Amino Acids								
Species	Ile	Leu	Lys	Mth	Tryp	Phe	His	Thre	Val	Arg	Cys	Tyr	Ala	Aa	Ga	Gly	Pro	Ser
*Acheta domesticus* A [44,47]	940	2050	1100	300	130	650	480	740	1070	1250	170	1000	1800	1720	2150	1040	1150	1020
*Acheta domesticus* L [43,45]	710	1270	1090	274	144	587	450	680	1050	1360	160	1100	1770	1390	2050	1060	1070	750
*Gryllus bimaculatus* A [47]	920	1650	1140	350	220	740	520	810	1360	1140	160	1170	1930	1970	2440	1240	1250	1050
*Tenebrio molitor* A [44]	1030	1960	1050	300	260	620	680	810	1500	1020	140	790	1810	1660	2280	2000	1500	980
*Tenebrio molitor* L [28,43,44]	835	1400	1070	400	216	654	559	770	1280	1380	163	1370	1640	1520	2130	1040	1300	960
*Zophobas mori* L [35,43]	881	1360	1070	255	203	740	600	780	1230	1290	175	1310	1440	1620	2440	950	1060	812
*Gonimbrasia belina* L [28,35,48]	1300	1830	1460	410	480	1350	600	1840	1120	2410	110	974	1300	2234	4120	1100	876	1210
*Bombyx mori* L [35,45]	290	430	440	110	60	250	260	250	350	380	80	300	360	610	900	510	310	340
*Pyralidae* L [35,45]	670	1240	920	440	140	600	360	590	840	820	210	880	1150	1490	1950	930	1240	1240
Mutton leg	773	1195	1267	381	196	621	425	727	785	1068	198	512	1026	1362	2289	919	725	650
Veal leg	826	1293	1349	413	174	660	551	688	853	1074	137	578	991	1514	2311	881	743	688
Horsemeat	1457	2129	2240	627	226	853	627	874	1122	1613	292	829	1212	1860	2735	964	896	943
Pork shoulder	821	1432	1483	487	235	699	584	966	927	1085	207	622	1064	1540	2542	921	659	620
Beef sirloin	997	1680	1844	560	232	911	706	951	1038	1309	265	746	1210	1862	3165	1007	783	835
Rabbit carcass	825	1277	1462	452	186	771	426	717	851	1063	213	611	1010	1595	2738	851	851	691
Goose carcass	264	493	515	144	84	254	162	268	287	390	41	206	365	547	917	357	257	232
Duck carcass	391	611	686	214	95	329	250	370	479	580	104	280	589	752	1445	656	438	372
Turkey breast	915	1419	2015	522	248	703	537	994	953	1237	121	618	1191	1901	3246	941	813	826
Turkey drumstick	797	1233	1758	452	217	607	468	865	826	1065	105	536	1029	1647	2823	775	681	717
Chicken breast	1251	1579	2022	631	360	772	941	911	1345	321	279	735	1441	2157	3505	1334	1028	886
Chicken drumstick	982	1240	1590	497	283	606	739	715	1057	1154	220	578	1134	1694	2756	1049	809	697

Column heading abbreviations are as follows: Ile—isoleucine, Leu—leucine, Lys—lysine, Mth—methionine, Cys—cystine, Phe—phenylalanine, Tyr—tyrosine, Thre—threonine, Tryp—tryptophane, Val—valine, Arg—arginine, His—histidine, Ala—alanine, Aa—aspartic acid, Ga—glutamic acid, Gly—glycine, Pro—proline, Ser—serine, A—adult insect, L—larval form.

**Table 4:** Fatty acids content of meat (g/100 g edible portion).

	Mutton Leg	Veal Leg	Horse Meat	Pork Shoulder	Beef Sirloin	Rabbit Carcass	Goose Carcass	Duck Carcass	Turkey Breast	Turkey Drumstick
SFA	7.41	0.98	0.89	2.74	1.68	2.59	3.00	4.73	0.22	1.12
C 10:0	0	0	0	0	0	0	0	0	0	0
C 12:0	0	0	0.01	0	0	0	0	0	0	0
C 14:0	0.76	0.10	0.08	0.10	0.11	0.16	0.06	0.08	0.01	0.04
C 15:0	0.08	0	0	0	0.03	0.04	0	0.02	0	0
C 16:0	3.40	0.53	0.64	1.57	0.88	1.79	2.21	3.55	0.16	0.82
C 17:0	0.14	0	0	0.00	0.05	0.05	0.01	0.03	0	0
C 18:0	2.94	0.35	0.16	1.05	0.61	0.55	0.71	0.91	0.05	0.26
C 20:0	0.02	0	0	0	0	0	0	0.12	0	0
MUFA	5.80	0.99	0.82	3.26	1.51	1.24	6.45	9.66	0.33	1.65
C 14:1	0.10	0.03	0	0	0.04	0	0	0.02	0	0
C 15:1	0	0	0	0	0.01	0	0	0	0	0
C 16:1	0.18	0.09	0.14	0.17	0.16	0.13	0.58	0.79	0.04	0.2
C 17:1	0.14	0	0	0	0.03	0	0	0.02	0	0
C 18:1	5.37	0.86	0.67	3.08	1.26	1.11	5.76	8.64	0.28	1.42
C 20:1	0	0	0.01	0	0.01	0	0.10	0.14	0.01	0.03
C 22:1	0	0	0	0	0	0	0	0.05	0	0
PUFA	0.73	0.13	0.62	0.58	0.11	2.03	2.68	2.64	0.15	0.77
C 18:2	0.35	0.09	0.28	0.52	0.08	1.25	2.22	2.48	0.13	0.63
C 18:3	0.35	0.03	0.25	0.07	0.03	0.59	0.46	0.14	0.01	0.07
C 20:3	0	0	0	0	0	0	0	0.00	0	0
C 20:4	0.03	0	0.04	0	0	0.11	0	0.02	0.01	0.07
C 20:5	0	0	0.01	0	0	0.08	0	0.00	0	0
PUFA/SFA	0.10	0.13	0.70	0.21	0.07	0.78	0.89	0.56	0.68	0.69
PUFA *n*-3	0.35	0.03	0.3	0.07	0.03	0.67	0.46	0.14	0.01	0.07
PUFA *n*-6	0.38	0.09	0.32	0.52	0.08	1.36	2.22	2.50	0.14	0.7
PUFA *n*-6/PUFA *n*-3	1.07	3.0	1.07	7.88	2.67	2.02	4.84	17.73	14.0	10.0
AI	0.98	0.84	0.67	0.51	0.81	0.74	0.27	0.32	0.42	0.40
TI	1.40	1.01	0.37	1.23	1.27	0.66	0.50	0.69	0.65	0.75
h/H	1.47	1.56	1.71	2.20	1.38	1.61	3.72	3.11	2.53	2.55

SFA—saturated fatty acids, MUFA—monounsaturated fatty acids, PUFA—polyunsaturated fatty acids, AI—atherogenic index, TI—thrombogenic index, h/H—hypocholesterolemic/hypercholesterolemic ratio.

**Table 5:** Fatty acids content of edible insects (g/100 g edible portion) [29,30,35,43,44,45,49].

	*Acheta* *domesticus* A [29,35,44]	*Acheta* *domesticus* L [43,44,45]	*Tenebrio molitor* L [30,35,44,45]	*Zophobas morio* L [35,43,44]	*Gonimbrasia belina* L [49]	*Galleria mellonella* L [43,44]
SFA	2.28	2.51	2.32	5.15	4.9	6.48
C 10:0	0.011	0.007	-	0.01	-	0.01
C 12:0	<0.02	0.007	<0.02	0.01	<0.1	<0.01
C 14:0	0.04	0.06	0.29	0.17	<0.1	0.04
C 15:0	<0.02	0.01	<0.02	0.04	-	<0.01
C 16:0	1.56	1.72	2.29	3.59	3.2	5.97
C 17:0	0.02	0.02	<0.02	0.075	-	0.01
C 18:0	0.58	0.65	0.39	1.15	1.7	0.41
C 20:0	0.04	0.03	0.03	0.03	<0.1	0.02
MUFA	1.694	1.77	2.51	4.46	1.7	8.35
C 14:1	0.02	-	-	0.01	-	<0.01
C 15:1	-	0.01	-	0.01	-	<0.01
C 16:1	0.09	0.09	0.35	0.14	0.1	0.40
C 17:1	<0.01	0,007	0.03	0.01	-	<0.01
C 18:1	1.54	1.64	5.39	4.28	1.6	7.90
C 20:1	0.02	0.01	-	0.02	-	0.01
C 22:1	0.014	0.007	-	0.01	-	<0.01
PUFA	2.43	4.28	5.85	5.46	5.4	1.88
C 18:2 *n*-6	2.29	2.07	-	2.64	1.6	1.76
C 18:3 *n*-3	0.06	0.35	-	0.38	3.7	0.11
C 20:3 *n*-6	0.02	0.01	-	0.01	-	<0.01
C 20:5 *n*-3	0.06	0.04	-	0.03	-	0.03
PUFA/SFA	1.07	1.70	2.52	1.06	1.10	0.28
PUFA *n*-3	0.12	0.39	-	0.41	3.7	0.14
PUFA *n*-6	2.31	2.08	-	2.65	1.6	1.77
PUFA *n*-6/PUFA *n*-3	19.25	5.33	18.44	6.46	0.43	12.64
AI	0.42	0.46	0.33	0.57	0.53	0.60
TI	0.90	0.74	0.26	0.99	0.39	1.17
h/H	2.45	2.30	3.82	1.95	2.0	1.63

A—adult insect, L—larval form.

**Table 6:** Mineral contents of edible insects and meat (mg/100 g edible portion) [28,29,30,35,36,43,44,50].

Minerals Species	Na	K	Ca	P	Mg	Fe	Zn	Cu	Mn	I
*Acheta domesticus* A [28,29,35,43,44]	163–178	347–390	99.6	899.3	55.1	5.46–8.83	6.71–11.0	0.62	1.15	0.15
*Acheta domesticus* L [43,44]	110.0	285.0	36.6	219.0	22.6	2.12	6.8	0.51	0.89	0.028
*Gryllus bimaculatus* A [35,36]	88.84	321.71	105.14	702.02	72.94	9.5	14.39	3.86	3.4	-
*Tenebrio molitor* A [30,35,44]	66.0	368.0	24.2	295.0	69	2,87	4.86	0.75	0.46	0.022
*Tenebrio molitor* L [29,30,35,43,44]	53.7	337.0	42.9	264–368	62–92	2.47	4.33–4.95	0.83	0.32	0.02
*Zophobas morio* L [35,43,44]	38.5	286.0	26.2	209.0	43.5	1.99	3.02	0.36	0.37	<0.01
*Gonimbrasia belina* L [35,50]	1024.0	1032.0	174.0	543.0	160	51.06	17.95	0.91	3.95	-
*Bombyx mori* L [35,44]	47.5	316–391	49.8–72.2	172–237	56.9–78.3	2.23	3.07	0.36	0.39	<0.01
*Pyralidae* L [35,44]	9.21–16.5	221.0	24.3	157–457	34.3	2.94	3.0	0.38	0.13	<0.01
Mutton leg	65.5	319.2	8.4	178.9	19.3	2.3	2.7	0.1	0.03	0.025
Veal leg	101.1	194.3	7.9	126.4	12.6	1.9	1.6	0.2	0.02	0.017
Horse meat	46.0	345.0	15.0	200.0	24.0	3.5	2.7	0.1	0.01	0.018
Pork shoulder	50.0	293.6	4.1	130.4	15.6	0.9	2.2	0.1	0.01	0.008
Beef sirloin	52.0	382.0	4.0	212.0	26.0	3.1	2.9	0.1	0.04	0.008
Rabbit carcass	34.0	304.2	15.0	144.6	20.5	2.1	1.7	0.1	0.03	0
Goose carcass	19.7	99.6	2.1	62.3	7.4	1.0	0.7	0.1	0.01	0.003
Duck carcass	42.2	154.9	5.1	95.4	9.0	1.3	0.9	0.1	0.02	0.008
Turkey breast	47.0	460.0	2.0	238.0	35.0	0.5	0.8	0.05	0.01	0.007
Turkey drumstick	96.0	364.0	8.0	214.0	27.0	1.2	2.8	0.1	0.02	0
Chicken breast	55.0	385.0	5.0	240.0	33.0	0.4	0.5	0.01	0.01	0
Chicken drumstick	91.0	334.0	8.0	215.0	26.0	0.7	1.4	0.1	0.01	0

Column heading abbreviations are as follows: Na—sodium, K—potassium, Ca—calcium, P—phosphorus, Mg—magnesium, Fe—iron, Zn—zinc, Cu—copper, Mn—manganese, I—iodine, A—adult insect, L—larval form.

**Table 7:** INQ values of selected minerals following the demand of women (31–50 years old, bodyweight 45 kg, moderate physical activity) **.

DRNI* Species	Na 1500 mg	K 3500 mg	Ca 1000 mg	P 700 mg	Mg 320 mg	Fe 18 mg	Zn 8 mg	Cu 0.9 mg	Mn 1.8 mg	I 150 µg
*Acheta domesticus* A	1.56	1.44	1.37	17.63	2.36	5.45	18.87	9.46	8.77	0.01
*Acheta domesticus* L	1.12	1.24	0.56	4.78	1.08	1.80	31.48	7.51	7.55	0.00
*Gryllus bimaculatus* A	1.04	1.61	1.84	17.55	3.99	6.96	12.98	8.65	33.06	0.00
*Tenebrio molitor* A	0.52	1.24	0.29	4.97	2.54	1.88	7.17	9.83	2.99	0.01
*Tenebrio molitor* L	0.30	0.82	0.36	4.00	2.05	1.17	4.93	7.84	1.51	0.00
*Zophobas morio* L	0.26	0.83	0.27	3.03	1.38	1.12	3.83	4.06	2.09	0.00
*Gonimbrasia belina* L	8.90	3.85	2.27	10.12	6.52	37.00	29.27	13.19	28.62	0.00
*Bombyx mori* L	0.37	1.19	0.72	3.43	2.48	1.46	4.51	4.70	2.55	0.00
*Pyralidae* L	0.07	0.48	0.19	2.95	0.82	1.25	2.87	3.23	0.55	0.00
Mutton leg	0.47	0.97	0.09	2.73	0.65	1.35	3.59	0.60	0.20	0.18
Veal leg	1.66	1.37	0.19	4.44	0.97	2.59	4.98	4.32	0.32	0.29
Horse meat	0.59	1.90	0.29	5.50	1.44	3.75	6.50	3.00	0.11	0.23
Pork shoulder	0.53	1.34	0.07	2.98	0.78	0.80	4.40	1.02	0.07	0.09
Beef sirloin	0.65	2.05	0.08	5.68	1.52	3.23	6.87	2.08	0.42	0.10
Rabbit carcass	0.39	1.48	0.26	3.52	1.09	1.94	3.69	2.24	0.30	0.00
Goose carcass	0.20	0.43	0.03	1.33	0.34	0.82	1.27	1.16	0.07	0.03
Duck carcass	0.30	0.47	0.05	1.44	0.30	0.79	1.20	1.05	0.11	0.05
Turkey breast	0.79	3.33	0.05	8.60	2.77	0.70	2.63	1.12	0.14	0.12
Turkey drumstick	1.34	2.18	0.17	6.42	1.77	1.40	7.43	1.87	0.23	0.00
Chicken breast	0.79	2.36	0.11	7.35	2.21	0.48	1.31	0.24	0.12	0.00
Chicken drumstick	1.02	1.60	0.13	5.16	1.37	0.65	2.94	1.49	0.09	0.00

Column heading abbreviations are as follows: Na—sodium, K—potassium, Ca—calcium, P—phosphorus, Mg—magnesium, Fe—iron, Zn—zinc, Cu—copper, Mn—manganese, I—iodine. ** Energy demand for women (aged between 31–50 years old, bodyweight 45 kg, moderate physical activity) is 2100 kcal/day. DRNI*—daily recommended nutrient intake based on Polish standards [42]. A—adult insect, L—larval form.

**Table 8:** Vitamin contents of edible insects and meat (per 100 g edible portion) [28,29,35,36,43,44].

Vitamins Species	A (µg/100 g)	E (mg/100 g)	B_1_ (mg/100 g)	B_2_ (mg/100 g)	PP (mg/100 g)	B_6_ (mg/100 g)	B_12_ (µg/100 g)	C (mg/100 g)
*Acheta domesticus* A [28,29,44]	6.53	2.26	0.04	3.41	3.84	0.23	0.53	3.0
*Acheta domesticus* L [28,43,44]	<30	0.64	0.24	1.66	3.28	0.17	0.87	1.8
*Gryllus bimaculatus* A [36]	-	-	0.36	1.91	3.10	-	-	-
*Tenebrio molitor* A [35,44]	<30	<0.34	0.1	0.85	5.64	0.81	0.56	5.4
*Tenebrio molitor* L [28,29,43,44,45]	16.9–29.0	1.31–1.9	0.18	0.81–1.21	4.07–4.65	0.81	0.47	1.8–9.9
*Zophobas morio* L [43,44]	<30	0.52	0.17	1.12	3.53	0.32	0.99	1.2–10.1
*Bombyx mori* L [44]	47.4	0.59	0.33	0.94	2.63	0.16	0.01	<1.0
*Pyralidae* L [44]	<30	0.89	0.23	0.73	3.75	0.13	0.12	<1.0
Mutton leg	44.52	0.28	0.13	0.18	4,37	0.13	0.84	0
Veal leg	23.70	0.24	0.14	0.22	5.14	0.24	1.03	0
Horse meat	30.00	0.52	0.14	0.27	4.50	0.50	3.10	0
Pork shoulder	0.00	0.33	0.49	0.23	4.86	0.25	0.57	0
Beef sirloin	11.00	0.20	0.12	0.26	5.54	0.25	1.40	0
Rabbit carcass	0.00	0.10	0.02	0.05	5.53	0.47	7.90	0
Goose carcass	12.30	0.08	0.05	0.01	2.62	0.24	0.12	0
Duck carcass	15.36	0.13	0.11	0.14	2.21	0.06	0.13	0
Turkey breast	9.00	0.02	0.04	0.15	4.92	0.59	0.70	0
Turkey drumstick	20.00	0.02	0.08	0.21	3.26	0.30	1.70	0
Chicken breast	6.00	0.30	0.09	0.15	12.44	0.55	0.40	0
Chicken drumstick	20.00	0.30	0.08	0.25	3.06	0.33	0.40	0

Column heading abbreviations are as follows: A—vitamin A, E—vitamin E, B1—thiamin, B2—riboflavin, PP—niacin, B6—pyridoxine, B12—cobalamin, C—vitamin C, A—adult insect, L—larval form.

**Table 9:** INQ values of selected vitamins following the demand of women (31–50 years old, bodyweight 45 kg, moderate physical activity) **.

DRNI* Species	Vit. A 700 µg	Vit. E 8 mg	Vit. B_1_ 1.1 mg	Vit. B_2_ 1.1 mg	Vit. PP 14 mg	Vit. B_6_ 1.3 mg	Vit. B_12_ 2.4 µm	Vit. C 75 mg
*Acheta domesticus* A	128.04	3.88	0.50	42.55	3.76	2.43	3.03	0.55
*Acheta domesticus* L	654.55	1.22	3.33	23.05	3.58	2.00	5.54	0.37
*Gryllus bimaculatus* A	-	-	5.73	30.39	3.88	-	-	-
*Tenebrio molitor* A	505.62	0.50	1.07	9.12	4.75	7.35	2.75	0.85
*Tenebrio molitor* L	278.74	1.71	1.39	7.42	2.59	5.30	1.68	0.49
*Zophobas morio* L	434.99	0.22	1.57	10.33	2.56	2.50	4.19	0.76
*Bombyx mori* L	795.48	0.87	3.52	10.04	2.21	-	0.59	0.16
*Pyralidae* L	327.63	0.85	1.60	5.07	2.05	1.45	0.38	0.10
Mutton leg	679.49	0.37	1.31	1.79	3.33	0.76	3.74	0
Veal leg	833.33	0.73	3.18	4.95	9.03	1.10	10.53	0
Horse meat	825.69	1.25	2.45	4.73	6.19	4.49	24.89	0
Pork shoulder	0	0.66	7.17	3.29	5.56	7.41	3.83	0
Beef sirloin	294.64	0.47	1.99	4.40	7.42	3.03	10.94	0
Rabbit carcass	0	0.22	0.37	0.73	6.73	3.61	56.09	0
Goose carcass	262.39	0.15	0.67	0.18	2.80	6.11	0.77	0
Duck carcass	231.51	0.17	1.09	1.39	1.66	2.73	0.56	0
Turkey breast	325.30	0.06	0.83	3.45	8.89	0.52	7.38	0
Turkey drumstick	600.00	0.05	1.49	4.07	4.89	11.48	14.88	0
Chicken breast	183.67	0.80	1.75	2.98	19.04	4.85	3.57	0
Chicken drumstick	480.00	0.63	1.22	3.76	3.67	9.07	2.80	0

Column heading abbreviations are as follows: A—vitamin A, E—vitamin E, B1—thiamin, B2—riboflavin, PP—niacin, B6—pyridoxine, B12—cobalamin, C—vitamin C. ** Energy demand for women (aged between 31–50 years old, bodyweight 45 kg, moderate physical activity) is 2100 kcal/day. DRNI*–daily recommended nutrient intake based on Polish standards [42]. A—adult insect, L—larval form.

## Data Availability

The datasets used and/or analyzed during the current study are available from the corresponding author on reasonable request.
